# Supplementary material for: Ultrasound Radiomics Nomogram to Diagnose Sub-Centimeter Thyroid Nodules Based on ACR TI-RADS
Source: Cancers (Basel). 2022 Oct 3;14(19):4826. doi: 10.3390/cancers14194826 (PMC9562658; doi:10.3390/cancers14194826)
Supplement: Supplementary file 1 [file cancers-14-04826-s001.zip › cancers-1790725-supplementary.pdf]

Table S1. Major packages of R software used in this study.

**Functions**	**R package**
LASSO regression	glmnet
Plot nomogram	rms
Plot bar diagrams	ggplot2
Decision curve analysis (DCA) and clinical impact curve (CIC)	rmda

Figure S1. A total of 10 radiomics features with non-zero coefficients were selected in the redundancy analysis and LASSO logistic regression model. A radiomics scoring formula was constructed, Rad-score = intercept + coefficient × radiomic features, and the radiomics scores of each patient were calculated.

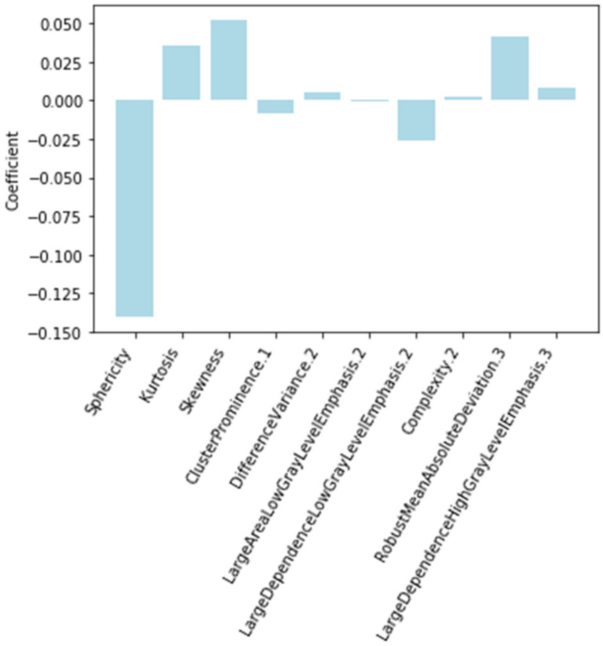

Rad-score formulas

Rad-score=4.13836918220756-4.221449889964304*Sphericity+0.017889011102550242*Kurtosis+0.07949908403034733*Skewness-1.0326270089944924e-08*ClusterProminence.1+9.743324586410937e-05*DifferenceVariance.2-0.008578683679038123*LargeAreaLowGrayLevelEmphasis.2-0.2636157249727618*LargeDependenceLowGrayLevelEmphasis.2+1.4096038564143772e-06*Complexity.2+0.004478017008477920 6*RobustMeanAbsoluteDeviation.3+2.370677481365933e-05*LargeDependenceHighGrayLevelEmphasis.3

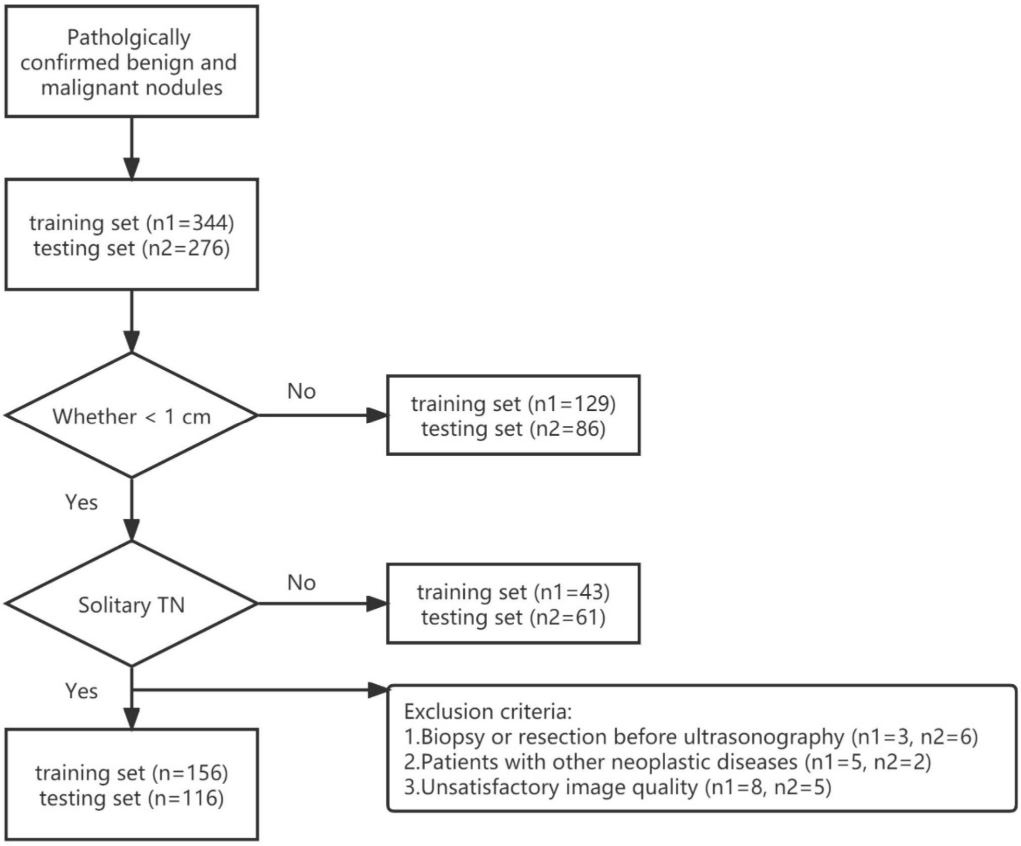

Figure S2. Shows a flowchart about selecting patients in more detail.

R code example

```
getwd() #
df_all <- read.csv("data.csv")
# 4.view the dimension of data
dimension <- dim(df_all)
dimension
col_name <- names(df_all)
col_name[1:100] # view top100 namesdf_all$label
# 5.training set/validation set
set.seed(1234)
training_index <- sample(1:200, 0.7 * 210, replace = F)
validation_index  <-  c(1:200)[-training_index]
# 6. check index
training_index
validation_index
# 7. set training/validation data、label
training_set <- df_all[training_index, ]
validation_set <- df_all[validation_index, ]
```

```
train_label <- training_set[, 1]
valid_label <- validation_set[, 1]
train_set_nolabel <- training_set[-1]

# 8. check the dimension
dim(training_set)
dim(validation_set)
# 9.feature selection: reduce redundancy
# 9.1 calculate p of normality test
norm_result <- apply(training_set, 2, function(x) shapiro.test(x)$p.value)
norm_feature <- training_set[which(norm_result >= 0.05)]
# 9.2 calculate r
cor_nor <- cor(norm_feature, method = "pearson")
cor_all <- cor(training_set, method = "spearman")

# 9.3 change matrix
num_nor <- dim(cor_nor)[1]
cor_all[1:num_nor, 1:num_nor] <- cor_nor
# 9.4 set 0
cor_all[upper.tri(cor_all)] <- 0
diag(cor_all) <- 0
# 9.5 get training_set after reduce redundancy
data_reduce = training_set[, !apply(cor_all, 2, function(x) any(abs(x) > 0.9))]
# 9.6 check new data
dim(data_reduce)
#View(data_reduce)
#10. feature selection: LASSo
#10.1 data preparation
cv_x <- as.matrix( data_reduce )
cv_y <- train_label
#10.2 LASS0 process
set.seed( 1 )
library("glmnet")
lasso_selection <- cv.glmnet(x = cv_x,
                             y = cv_y,
                             family = "binomial",
                             type.measure = "deviance",# deviance
                             alpha = 1,
                             nfolds = 5)
#10.3 two pictures for lasso
par(font.lab=2,mfrow=c(2,1),mar=c(4.5,5,3,2))
plot(x=lasso_selection,las=1,xlab="Log(lambda)") # Fig 1
nocv_lasso <- glmnet(x=cv_x,y=cv_y,family="binomial",alpha = 1)
plot(nocv_lasso,xvar="lambda",las=1,lwd=2,xlab="Log(lambda)") # Fig 2
```

```
abline(v=log(lasso_selection$lambda.min),lwd=1,lty=3,col="black")
#10.4 bget cofficient
coefPara <- coef(object=lasso_selection,s="lambda.1se")
lasso_values <- as.data.frame(which(coefPara !=0,arr.ind = T))
lasso_names <- rownames(lasso_values)[-1]
Lasso_coef <- data.frame(Feature =rownames(lasso_values),
                         Coef = coefPara[which(coefPara !=0,arr.ind = T)]
Lasso_coef
##
##
##
library(rmda)
getwd() #

Data<-read.csv('CIC.csv',sep = ',')

## Warning in decision_curve(chdfate ~ scl, data = Data, family =
## binomial(link = "logit"), : 33 observation(s) with missing data removed
Nomogram <-decision_curve(label~R,
                         data = Data,family = binomial(link ='logit'),
                         thresholds = seq(0,1, by = 0.01),
                         confidence.intervals= 0.95,
                         study.design = 'cohort' )
## Warning in decision_curve(chdfate ~ scl + sbp + dbp + age + sex, data
List<- list(Nomogram)
plot_decision_curve(List,
                    curve.names=c('Clinical','Rad-Score','Nomogram'),
                    cost.benefit.axis =FALSE,col= c('blue','green','red'),
                    confidence.intervals=FALSE,
                    standardize = FALSE)
#绘制（Clinical Impact Curve）
plot_clinical_impact(Nomogram,population.size= 1000,#
                     cost.benefit.axis = T,#
                     n.cost.benefits= 8,
                     col =c('red','blue'),
                     confidence.intervals= F,
                     ylim=c(0,1000),
                     legend.position="topright")
```
